# Post-traumatic Stress and Depressive Symptoms Among Adolescents After the 2015 Earthquake in Nepal: A Longitudinal Study

**DOI:** 10.1007/s10578-021-01136-3

**Published:** 2021-02-19

**Authors:** Sanju Silwal, Roshan Chudal, Ragnhild Dybdahl, Lauri Sillanmäki, Lars Lien, Andre Sourander

**Affiliations:** 1 Department of Child Psychiatry, Research Centre for Child Psychiatry, University of Turku, Turku, Finland; 2 Department of Social Work, Child Welfare and Social Policy, OsloMet University, Oslo, Norway; 3 Faculty of Psychology, Center for Crisis Psychology, University of Bergen, Bergen, Norway; 4 Norwegian National Advisory Unit On Concurrent Substance Abuse and Mental Health Disorders, Innlandet Hospital Trust, Brumunddal, Norway; 5 Department of Public Health, Inland Norway University of Applied Sciences, Elverum, Norway; 6 Department of Child Psychiatry, Turku University Hospital, Turku, Finland; 7 INVEST Research Flagship, University of Turku, Turku, Finland; 8 Department of Clinical Science, Faculty of Medicine, Research Centre for Child Psychiatry, University of Turku, Lemminkäisenkatu 3 / Teutori (3rd. floor), 20014 Turku, Finland

**Keywords:** Adolescent, Earthquake, Longitudinal study, Post-traumatic stress disorder, Depression

## Abstract

Few longitudinal studies have focused on mental health problems among adolescents after earthquakes. We investigated changes in post-traumatic stress symptoms (PTSS) and depressive symptoms from 18 to 31 months after the 2015 earthquake in Nepal and explored potential risk factors associated with the change in psychiatric symptoms. This study comprised of 515 adolescents, aged 11–17 years from two earthquake-affected areas, one severely affected than the other. The psychiatric symptoms were assessed using the standardized Child Post-traumatic Stress Disorder Scale and the Depression Self-Rating Scale. No significant change was observed in the prevalence of PTSS and depressive symptoms from 18 to 31 months after the earthquake. Living in severely affected area and exposure to trauma after the earthquake were associated with adolescents who developed chronic or delayed PTSS and depressive symptoms. The study findings highlight the need for disaster preparedness and early interventions that strengthen support at various levels.

**Supplementary Information:**

The online version contains supplementary material available at (10.1007/s10578-021-01136-3).

## Introduction

About 70% of individuals are exposed to traumatic events in their lifetime, and 7% experience natural disasters[1]. Exposure to natural disasters, like other traumas, have shown to increase negative outcomes to mental health; most commonly reported are post-traumatic stress disorder (PTSD) and depression [2–4]. Studies have shown that in the aftermath of a disaster, children and adolescents show a greater risk of severe impairment compared to adult survivors [5]. The prevalence of PTSD among children and adolescents ranged from 1.0 to 60.0% and depression from 1.6 to 33.0%[3]. These disorders can have long-lasting effects on the physical and psychological development and quality of life [6–9].

Numerous studies have shown an increased risk for psychiatric disorders even after years of exposure to natural disasters [10, 11]. In a study by Piyasil et al. [12], the prevalence rate of PTSD was 2.7% in children after 5 years of tsunami exposure. Likewise, a study by Lai et al. [13] showed 7.0% of children with PTS and 11.0% with depression 15 months post-Hurricane Ike. Most longitudinal studies among adolescents after natural disasters indicated a gradual decline of psychiatric disorders over time [14–21]; however, persistence of symptoms was reported in some studies [22–24]. In contrast, an increase in the prevalence of PTSD and depression was reported in a study by Liu et al. [25] conducted at 6 and 12 months after the 2008 Sichuan earthquake.

There is great variability in the prevalence rates after earthquake exposure [14, 15, 17, 19, 23, 26]. In a study by Du et al. [27], the prevalence of PTSD in adolescents after 36 months of the Wenchuan earthquake was 11.2% and depression was 30.3%. While in another study, PTSD prevalence was 15.7% and depression 21.6% among adolescents 30 months after the Ya’an and Wenchuan earthquake [21]. These varied prevalence rates could be due to the use of various methodologies and assessments conducted at different time-points. Thus, it is important to evaluate psychiatric symptoms in uniform time-points after disasters, which might help us to comprehend the process of psychopathology over time.

Loss of family members, being female, witnessed death, being seriously injured and property damage were important risk factors reported by adolescents after earthquake exposure [15, 17, 21]. Various degrees of earthquake exposure, such as severity and closeness to the epicenter, have shown to influence the development of psychiatric symptoms [28]. However, it remains unclear how the severity of earthquake exposure influences the stability of mental health symptoms over time. Exposure to multiple traumatic events has been associated with high levels of PTSD in adults [29, 30] but has been rarely examined in adolescents [31].

The main aim of the present study was to analyze changes in PTSS and depressive symptoms among adolescents 18 to 31 months after the 2015 Nepal earthquake. Based on the previous research [22], we hypothesized that PTSS and depressive symptoms experienced by adolescents after the earthquake would decrease over time. The second aim of the present study was to investigate potential risk factors that may influence psychiatric symptoms over time.

## Methods

### The Present Study

Nepal is a small landlocked country in South Asia, with a population of almost 28.6 million [32]. On 25 April 2015, a 7.8 magnitude earthquake hit Nepal, causing 9000 deaths, 22,000 injuries and 706 billion NPR (7 billion US dollars) worth of damage [32]. We examined the impact of the earthquake on the mental health of adolescents living in two earthquake-affected areas, Sindhupalchok and Kathmandu districts. In Sindhupalchok, the earthquake severely affected the district, causing 3570 deaths and over 605 million NPR (six million US dollars) worth of damage. In Kathmandu, the earthquake affected the district to a slightly less extent, with 1233 deaths and 302 million NPR (three million dollars) worth of damage. A preliminary baseline study was conducted 18 months after the earthquake among adolescents from these two districts [33]. The findings demonstrated that adolescents experienced PTSS and depressive symptoms after the earthquake and the prevalence rates of both symptoms were higher in adolescents living in a severely affected area.

### Participants

This longitudinal study included adolescents who had experienced the 2015 earthquake in Nepal. Assessments were conducted at 18 (baseline) and 31 months (follow-up) after the earthquake. In the preliminary baseline study, 893 adolescents aged 11–17 years from grade 7–10 were enrolled from Sindhupalchok and Kathmandu districts. The design and methods of the present study are given below in a summarized form; however, a detailed description of the preliminary baseline study was reported in a previous study [33]. All the schools who had participated in the preliminary baseline study agreed to participate in the follow-up study. A total of 558 participants were available for the follow-up study. Out of 558, 32 adolescents were not in attendance at school to participate and 11 adolescents did not fully complete their follow-up questionnaires. This resulted in a final sample for the present study of 515 adolescents. Approximately 335 adolescents who participated in the preliminary baseline study were unavailable for follow-up as they had either graduated or moved to a different school. The participants that were lost at follow-up did not differ significantly with regard to demographic factors, earthquake-related factors, or their psychiatric symptoms.

### Procedure

Schools included in the preliminary baseline study were contacted to determine their willingness to participate in the follow-up study. The questionnaire was distributed in the classroom in the local Nepali language, and participants completed the questionnaires in about 45 min. The researcher (SS) and class teachers helped with the data collection. To appreciate the participation of the adolescents in the study, they were given compensation worth NPR 50. The study was approved by the Nepal Health Research Council and the Ethics Committee of the University of Turku, Finland. The District Education office in Nepal and the respective schools gave their approval for their students to participate in the study. Only after receiving written informed consent from parents, adolescents were included in the study. Information on parental demographic information was completed by adolescents.

### Measures

The demographic factors included sex, age, area of location, ethnicity, parents’ education. Sex of the participants was assessed as (male, female, and others), and age was categorized into ≤ 15 years and > 15 years. The area of location had two categories Sindhupalchok and Kathmandu. Ethnicity was classified into three categories: Brahmin or Chhetri, Janjati, and Dalit. Maternal and paternal education was classified as no education, secondary education (at least ten years of formal education), and higher education (at least twelve years of formal education).

Pre-earthquake trauma exposure was assessed using binary yes/no questions (i.e., did you experience trauma before the 2015 earthquake). We gave some examples of traumatic events in the question itself to ensure that the participants understood the meaning of traumatic events. If participants answered yes, then they were asked to select the type of traumatic events they had experienced from a structured list. The list included the following types of traumatic events, e.g., accidents, natural disaster, community violence, domestic violence, loss of family members/relatives and sexual abuse. The degree to which adolescents were exposed to trauma after the earthquake was assessed in a similar fashion to their pre-earthquake trauma exposure, using an initial binary yes/no response and then asking participants to select trauma types they have experienced after the earthquake. Adolescents exposed to trauma during the earthquake were operationalized in regard to yes/no binary responses on two factors: being trapped or wounded during the earthquake. House damage by the earthquake was identified on a four-point Likert scale as not at all, mild, moderate (home inhabitable), and severe (home uninhabitable).

#### Post-traumatic Stress Symptoms

Post-traumatic Stress Symptoms were evaluated using the Child Posttraumatic Stress Disorder Scale (CPSS) [34]. This instrument has frequently been used to assess children exposed to trauma after major disasters and catastrophic violence, and it has shown good psychometric properties in the Nepalese context [35]. It consists of 17 items (e.g. ''having bad dreams or nightmares'') and children rate items on a four-point Likert scale based on the frequency over the past week. The individual questions are scored as follows: zero = not at all, one = once in a week or less/once in a while, two = 2–4 times a week/ half the time, and three = five or more times a week almost always. The total score ranges from zero to 51. The cut-off score of ≥ 20 was used based on the validation study in Nepal[34]. In this study, Cronbach’s α values were 0.91 at 18 months and 0.91 at 31 months.

#### Depression

Depressive symptoms were assessed by the Depression Self-Rating Scale (DSRS), which is widely used to measure depression in children and adolescents between 8 and 14 years of age [36, 37]. It contains 18 items (e.g. ''I sleep very well'') and is based on a three-point Likert scale: zero = mostly, one = sometimes and two = never. The total score ranges from zero to 36, with a clinical cut-off score of ≥ 14 [37]. The DSRS has previously been validated in Nepalese children and cut-off score of ≥ 14 [35]. Cronbach’s α values for this study were 0.52 at 18 months and 0.56 at 31 months.

### Statistical Analysis

Descriptive analyses were conducted to examine participants’ demographic factors, trauma exposures and psychiatric symptoms. At both time points, 18 months and 31 months, PTSS and depressive symptoms were categorized based on the clinical cut-off score. The change in PTSS and depressive symptoms was estimated using generalized estimating equation (GEE) technique. Then, to estimate the association between potential risk factors and PTSS and depressive symptoms at 31 months after the earthquake, a binary logistic regression analysis was performed. Our analysis of those who were lost to follow-up revealed that there was no significant difference between demographic factors and trauma exposure measures and in scores of PTSS and depressive symptoms.

To observe symptomatic changes, the sample was divided based on cut-off scores into four groups. These groups were resilience (no symptoms at both time points), recovery (moderate/severe symptoms at baseline and no symptoms at follow-up), delayed (no symptoms at baseline, followed by elevated symptoms at follow-up) and chronic (moderate or severe symptoms at both time points) [38]. For PTSS, the resilience group was characterized by the participants’ CPSS scores being below the cut-off of 20 at both time points. The recovery group had CPSS scores equal to or above the cut-off of 20 at 18 months, but below the cut-off at 31 months. The delayed group was characterized by participants’ CPSS scores being below the cut-off of 20 at 18 months but above the cut-off at 31 months. The chronic group was characterized by participants’ whose CPSS scores were equal to or above the cut-off of 20 at both time points.

Similarly, we identified four groups with regard to depressive symptoms changes over time. The resilience group comprised participants’ with DSRS scores below the cut-off of 14. The recovery group comprised participants’ whose DSRS scores were equal to or above the cut-off of 14 at 18 months but below the cut-off at 31 months. The delayed group was characterized by participants’ DSRS scores being below the cut-off of 14 at 18 months but above the cut-off at 31 months. The chronic group included participants’ whose DSRS scores were equal to or above the cut-off of 14 at both time points.

We then used multinomial logistic regression to examine the predictors for four symptomatic groups identified in the change in PTSS and depressive symptoms among adolescents. Odds ratios (OR) were used to estimate the strength of the associations. Independent factors with a statistical significance of p < 0.05 were included in the logistic multiple predictor models and ninety-five percent confidence intervals (95% CI) were calculated for the OR values. All the statistical analyses were performed with Statistical Analysis System (SAS) software, version 9.4 [39].

## Results

### Sample Characteristics

Table 1 shows the demographics factors, trauma exposures and psychiatric symptoms by districts at 31 months after the earthquake. Of the total sample (N = 515), 258 were from Sindhupalchok (58.1% female) with a mean age of 15.13 years (SD = 1.18), and 257 were from Kathmandu (48.6% female) with a mean age of 14.56 years (SD = 0.98). There were significant differences between the two districts in terms of sex, age, ethnicity, pre-earthquake trauma exposure, trauma exposure after the earthquake, trapped/wounded and house damage. The number of fathers and mothers with higher education were 44.8% and 25.0%, respectively, in Kathmandu, and 30.1% and 16.6% in Sindhupalchok. Of the participants, more adolescents in Sindhupalchok had experienced trauma before the earthquake (52.1%) and had experience trauma after the earthquake (52.1%) compared to adolescents living in Kathmandu (27.7% and 27.7%, respectively). There were significant differences between Sindhupalchok and Kathmandu districts in the prevalence of PTSS and depressive symptoms. The prevalence of PTSS and depressive symptoms were higher, 32.7% and 46.9% in Sindhupalchok compared to 9.9% and 22.6% in Kathmandu district, respectively.

**Table 1:** Demographic factors, trauma exposures and PTSS and depressive symptoms at 31 months after the earthquake, stratified by area of location

Characteristics	No (%) of participants				Total		P-value^a^
	Sindhupalchok (N = 258)		Kathmandu N (= 257)				
	n	%	n	%	n	%	
Sex							0.031
Female	150	58.1	125	48.6	275	53.4	
Male	108	41.9	132	51.4	240	46.6	
Age (years)							< 0.001
≤ 15	97	37.0	47	18.4	144	28.1	
> 15	160	62.3	209	81.6	369	71.9	
Ethnicity							< 0.001
Brahmin/Chhetri	106	41.3	162	64.0	268	52.6	
Janjati	131	50.8	87	34.4	218	42.8	
Dalit	20	7.8	4	1.6	24	4.7	
Mother’s education							< 0.001
No education	89	35.2	39	15.2	128	25.2	
Secondary	122	48.2	153	59.8	275	54.0	
Higher secondary and above	42	16.6	64	25.0	106	20.8	
Father’s education							< 0.001
No education	48	18.8	24	9.3	72	14.0	
Secondary	131	51.2	118	45.9	249	48.5	
Higher secondary and above	77	30.1	115	44.8	192	37.4	
Pre-earthquake trauma exposure							< 0.001
Yes	127	52.1	70	27.7	197	39.6	
No	117	47.9	183	72.3	300	60.4	
Trauma exposure after earthquake							< 0.001
Yes	127	52.1	70	27.7	191	38.4	
No	117	47.9	183	72.3	306	61.6	
Trapped/Wounded							0.011
Yes	41	15.9	22	8.6	63	12.2	
No	217	84.1	235	91.4	452	87.8	
House damage							< 0.001
Not at all	31	12.0	129	50.2	160	31.1	
Mild	68	26.4	81	31.5	149	28.9	
Moderate	26	10.1	9	3.5	35	6.8	
Severe	133	51.6	38	14.8	171	33.2	
PTSS (≥ 20 CPSS scores)	81	32.7	24	9.9	105	20.4	< 0.001
Depressive symptoms (≥ 14 DSRS scores)	117	46.9	54	22.5	171	33.2	< 0.001

*CPSS* Child Post-traumatic Stress Disorder Scale, *DSRS* Depression Self-Rating Scale

^a^Pearson Chi-square

### Stability of PTSS and Depressive Symptoms from 18 to 31 Months After the Earthquake

Table 2 shows the change in the prevalence of PTSS and depressive symptoms at 18 and 31 months after the earthquake. There was no significant change in the prevalence of PTSS and depressive symptoms from 18 to 31 months after the earthquake, p > 0.05. The change in PTSS and depressive symptoms were not significant based on sex and area of location, p > 0.05.

**Table 2:** PTSS and depressive symptoms assessed at 18 and 31 months after the earthquake

	PTSS			Depressive symptoms		
	18 months n (%)	31 months n (%)	P-value^a^	18 months n (%)	31 months n (%)	P-value ^a^
Overall	134 (26.0)	105 (20.4)	0.10	193 (37.5)	171 (33.2)	0.69
Sex						
Female	88 (65.7)	64 (60.9)	0.065	116 (60.1)	102 (59.7)	0.291
Male	46 (34.3)	41 (39.1)	0.667	77 (39.9)	69 (40.4)	0.612
Area of location						
Sindhupalchok	100 (74.6)	81 (77.1)	0.118	119 (61.7)	117 (68.4)	0.047
Kathmandu	34 (25.4)	24 (22.9)	0.507	74 (38.3)	54 (31.6)	0.097

^a^Binary logistic regression with generalized estimating equation (GEE). Summary of separate GEE models

### Predictors of PTSS and Depressive Symptoms

Adolescents from the severely affected area (Sindhupalchok) had increased odds of developing PTSS (OR 2.62, 95% CI 1.35–5.11) and depressive symptoms (OR 1.95, 95% CI 1.12–3.33) compared to adolescents from Kathmandu (Table 3). The odds for PTSS and depressive symptoms were higher if the adolescent had experienced trauma after the earthquake (OR 2.83, 95% CI 1.57–5.09) and (OR 2.80, 95% CI 1.72–4.58).

**Table 3:** Associations between demographic factors and trauma exposures, and PTSS and depressive symptoms, 31 months after earthquake

	PTSS		Depressive symptoms	
	Single predictor model	Multiple predictors model	Single predictor model	Multiple predictors model
	OR (95% CI)	OR (95% CI)^a^	OR (95% CI)	OR (95% CI)^b^
Sex				
Female	1.49 (0.97–2.33)		1.44 (0.99–2.10)	
Male	Ref		Ref	
Age (years)				
≤ 15	Ref		Ref	Ref
> 15	2.07 (1.31–3.26) **	1.41 (0.83–2.38)	2.19 (1.45–3.30) ***	1.52 (0.96–2.41)
Ethnicity				
Brahmin/Chhetri	Ref		Ref	Ref
Janjati	1.33 (0.84–2.09)	1.08 (0.65–1.81)	1.28 (0.87–1.89)	1.06 (0.68–1.63)
Dalit	4.17 (1.73–10.02) **	2.25 (0.81–6.29)	2.37 (1.00–1.88) *	0.95 (0.35–2.56)
District				
Sindhupalchok	4.43 (2.69–7.28) ***	2.62 (1.35–5.11) **	3.05 (2.06–4.52) *	1.95 (1.12–3.33) *
Kathmandu	Ref		Ref	Ref
Mother’s education				
No education	Ref		Ref	
Secondary	0.96 (0.50–1.83)		0.89 (0.52–1.53)	
Higher secondary and above	0.9 (0.53–1.52)		0.74 (0.47–1.16)	
Father’s education				
No education	Ref		Ref	
Secondary	0.91 (0.48–1.73)		0.76 (0.43–1.31)	
Higher secondary and above	0.92 (0.48–1.78)		1.05 (0.58–1.84)	
Pre-earthquake trauma exposure				
Yes	3.23 (2.08–5.19) ***	1.57 (0.89–2.75)	2.55 (1.73–3.75) ***	1.32 (0.82–2.12)
No	Ref	Ref	Ref	Ref
Trapped/wounded				
Yes	1.50 (0.82–2.76)		1.12 (0.64–1.97)	
No	Ref		Ref	
House damage				
Not at all	Ref	Ref	Ref	Ref
Mild	1.47 (0.81–2.65)	0.90 (0.45–1.83)	1.23 (0.75–2.02)	0.87 (0.49 -1.53)
Moderate	1.36 (0.53–3.48)	0.60 (0.20–1.79)	1.67 (0.74–3.77)	0.82 (0.32–2.12)
Severe	1.85 (1.26–3.23) *	0.77 (0.70–1.45)	1.85 (1.13–2.89) *	0.95 (0.52–1.74)
Trauma exposure after earthquake				
Yes	5.32 (3.29–8.61) ***	2.83 (1.57–5.09) **	4.35 (2.91–6.51) **	2.80 (1.72–4.58) ***
No	Ref		Ref	

N = 515*.* Results of binary logistic regression analyses

*OR* odds ratio, *CI* confidence interval

*p < 0.05, **p < 0.01, ***p < 0.001

^a^Regression controlled for age, ethnicity, area of location, pre-earthquake trauma exposure, house damage and trauma exposure after earthquake

^b^Regression controlled for age, ethnicity, area of location, pre-earthquake trauma exposure, house damage and trauma exposure after earthquake

The detailed description of four groups (resilience, recovery, delayed and chronic) of symptomatic changes in participants are shown in Supplementary Tables 1 and 2. Table 4 shows the predictors of PTSS and depressive symptomatic change groups. When the resilience group was used as the reference category, adolescents in the delayed group were more likely from the severely affected area (OR2.58, 95% CI 1.04–6.42) and who had experienced trauma after the earthquake (OR 3.62, 95% CI 1.08–8.19). The risk for chronic PTSS was increased in female adolescents (OR 2.14, 95% CI 1.08–4.25), adolescents from the severely affected area (OR 4.34, 95% CI 1.76–10.75) and those exposed to trauma after the earthquake (OR 3.37, 95% CI 1.52–7.47).

**Table 4:** Multiple predictor multinomial logistic regression analysis results predicting PTSS and depressive symptoms groups

	PTSS^a^			Depressive symptoms^b^		
	Recovery	Delayed	Chronic	Recovery	Delayed	Chronic
	OR (95% CI)	OR (95% CI)	OR (95% CI)	OR (95% CI)	OR (95% CI)	OR (95% CI)
Sex						
Female	1.40 (0.79–2.47)	0.85 (0.43–1.68)	2.14 (1.08–4.25) *	0.92 (0.54–1.56)	0.97 (0.56–1.67)	1.86 (1.05–3.31) *
Male	Ref	Ref	Ref	Ref	Ref	Ref
Age						
≤ 15 years	Ref	Ref	Ref	Ref	Ref	Ref
> 15 years	0.74 (0.39–1.43)	1.90 (0.94–3.85)	0.92 (0.44–1.91)	0.76 (0.39–1.46)	1.28 (0.69–2.36)	1.77 (0.96–3.24)
Ethnicity						
Brahmin/Chhetri	Ref	Ref	Ref	Ref	Ref	Ref
Janjati	0.62 (0.34–1.11)	1.21 (0.59–2.48)	0.75 (0.37–1.49)	1.39 (0.82–2.40)	1.13 (0.64–1.98)	1.18 (0.66–2.09)
Dalit	1.39 (0.34–5.7)	2.35 (0.54–10.19)	2.82 (0.74–10.79)	1.94 (0.49–7.62)	1.20 (0.30–4.76)	1.05 (0.29–3.86)
Area of location						
Sindhupalchok	2.98 (1.45–6.09) **	2.58 (1.04–6.42) *	4.34 (1.76–10.75) **	1.53 (0.81 -2.92)	1.79 (0.89–3.62)	2.61 (1.25–5.44) **
Kathmandu	Ref	Ref	Ref	Ref	Ref	Ref
Pre-earthquake trauma exposure						
Yes	1.31 (0.69–2.47)	1.68 (0.78–3.63)	1.69 (0.80–3.55)	1.58 (0.86–2.92)	1.06 (0.57–2.00)	2.09 (1.12–3.94) *
No	Ref	Ref	Ref	Ref	Ref	Ref
Trapped/wounded						
Yes				2.42 (1.14–5.15) *	0.85 (0.31–2.33)	1.61 (0.71–3.66)
No				Ref	Ref	Ref
House damage						
Not at all	Ref	Ref	Ref	Ref	Ref	Ref
Mild	0.99 (0.45–2.19)	0.72 (0.28–1.87)	1.13 (0.44–2.91)	1.02 (0.52–2.01)	0.68 (0.34–1.39)	1.18 (0.53–2.62)
Moderate	1.17 (0.37–3.71)	0.98 (0.26–3.75)	0.34 (0.06–1.93)	1.01 (0.31–3.28)	0.75 (0.22–2.60)	0.91 (0.25–3.26)
Severe	1.27 (0.5 6–2.87)	0.65 (0.24–1.79)	1.09 (0.41–2.88)	1.10 (0.53–2.32)	0.75 (0.34–1.65)	1.41 (0.61–3.27)
Trauma exposure after earthquake						
Yes	2.31 (1.19–4.45) *	3.62 (1.60–8.19) ***	3.37 (1.52–7.47) **	0.50 (0.25–1.02)	2.42 (1.26–4.62) **	2.09 (1.07–4.09) *
No	Ref	Ref	Ref	Ref	Ref	Ref

N = 515. Reference group of PTSS and depressive symptoms = Resilient group

*OR* odds ratio, *CI* confidence interval, *Ref.* Reference

*p < 0.05, **p < 0.01, ***p < 0.001

^a^Regression controlled for sex, age, ethnicity, area of location, pre-earthquake trauma exposure, house damaged and trauma exposure after earthquake

^b^Regression controlled for sex, age, ethnicity, area of location, pre-earthquake trauma exposure, trapped/wounded, house damaged and trauma exposure after earthquake

In the depressive symptom group, as shown in Table 4, delayed depressive symptoms were observed among adolescents who had experienced trauma after the earthquake (OR 2.42, 95% CI 1.26–4.62). In the chronic group, female adolescents had increased odds of developing chronic depressive symptoms (OR 1.86, 95% CI 1.05–3.31). The odds also increased if adolescents were from the severely affected area (OR 2.61, 95% CI 1.25–5.44), had experienced pre-earthquake trauma (OR 2.09, 95% CI 1.12–3.94) and trauma after the earthquake (OR 2.09, 95% CI 1.07–4.09).

## Discussion

This is the first longitudinal study that examined PTSS and depressive symptoms experienced by adolescents exposed to the 2015 Nepal earthquake. The study had three major findings. First, the study showed no significant change in the prevalence of PTSS and depressive symptoms among adolescents from 18 to 31 months after the earthquake. Second, living in severely affected area and exposure to trauma after the earthquake were associated with PTSS and depressive symptoms at 31-month follow-up. Third, when the sample was divided based on the stability of symptoms into four groups (resilience, recovery, delayed and chronic), living in severely affected area and trauma exposure after the earthquake predicted those who developed chronic PTSS or depressive symptoms across time, as well as those who developed delayed PTSS or depressive symptoms.

In the present study, the prevalence rates of PTSS and depressive symptoms persisted over time, which is consistent with the previous studies [22, 23]. The persistence of symptoms could be related to the secondary stressors that adolescents face after the earthquake, such as property damage and poverty, which could have a cumulative impact. Conversely, adolescents with PTSS and depressive symptoms may find it difficult to continue normal school and social functioning, which in turn hamper coping with the symptoms.

The prevalence rates in the present study were higher compared to previous longitudinal studies with similar follow-up time [15, 22, 40]. This could be attributed to differences in assessment tools, study populations and the degree of trauma exposures. For example, 8.8% of adolescents reported severe post-traumatic stress symptoms 32 months after the Parnitha 1999 Athens earthquake using a self-report questionnaire [15], while 15.7% of adolescents had PTSD at 30 months using psychiatric interview [21]. On the other hand, sites of data collection have varied widely across studies, from schools [14] to community samples [22], and data gathered among populations close to the epicenter [15] or far from it [23]. Similarly, depression rates were higher in our study compared to previous studies where assessments were conducted after 30 months of the earthquake [21, 23]. It is possible that the high prevalence rates in our study could be linked to lower socioeconomic status, and thus, include more risk factors associated with poverty and lower educational levels that increased secondary stressors.

The findings showed that adolescents living in a severely affected area had increased risk for psychiatric symptoms at 31-months follow up and those developing delayed and chronic PTSS or depressive symptoms. The probable explanation could be that adolescents in severely affected area had a high degree of exposure to the earthquake in terms of deaths and destructions. The grief and despair induced by the earthquake could have increased the risk of developing psychiatric symptoms, even years after the disasters [28, 40]. On the other hand, this area is characterized by low socioeconomic development and low access to health care compared to Kathmandu, which is the capital city. Furthermore, adolescents with PTSS have reported avoiding sources of social support [41], which could have increased their vulnerability in the long run.

The study findings showed a dose–response effect of earthquake exposure to psychiatric symptoms. Adolescents who had experienced additional trauma after the earthquake were more likely to develop PTSS and depressive symptoms, which is in line with previous studies [42, 43]. These adolescents were more likely to develop delayed and chronic PTSS and depressive symptoms. This is an important finding and has rarely been examined in previous research. Studies have shown that additional trauma has a cumulative effect on post-traumatic reactions that exacerbate the symptoms [29, 31]. There is a dose–response relationship of multiple traumas impairing the interpersonal behavior and coping strategies [42]. More studies are needed to investigate the role of additional trauma exposure in long-term disaster responses. In addition, female adolescents were more likely to develop chronic symptoms, consistent with previous studies [44–47]. These gender differences may be due to variation in cognitive appraisal styles [48] and biological [49, 50] responses to traumatic events. It is also possible that girls are more likely to experience severe traumatic events such as violence and sexual abuse and receive poorer health care [51].

The study had some limitations that need to be considered when interpreting the results. First, the study was restricted to self-reports, while clinical interviews or observations would have provided additional information. Second, the loss of participants from the baseline to the follow-up study could have affected the findings. However, in the attrition analysis, there was no significant difference in the demographic factors, trauma exposures and psychiatric symptoms at baseline between those who participated in the follow-up and dropouts. Third, Cronbach's α for DSRS was low; however, DSRS results in our study are in line with changes in PTSS scores, which shows some kind of internal consistency. Fourth, the role of support and positive coping has been found to predict post-traumatic stress symptoms [44] but was not investigated in this study.

## Summary

This study contributes to the limited longitudinal studies that have examined mental health consequences in adolescents after earthquakes. Findings suggested that there was no significant change in the prevalence of PTSS and depressive symptoms among adolescents 18 and 31 months after the 2015 Nepal earthquake. Adolescents living in severely affected area and exposure to trauma after the earthquake were at a greater risk for developing delayed and chronic PTSS and depressive symptoms. As results suggested that adolescents exposed to the earthquake were at greater risk for developing delayed and chronic symptoms, it is important to design and implement long-term mental health interventions to identify them and provide early and preventative interventions. The study findings reinforce the importance of disaster preparedness and interventions that need to strengthen support at various levels. Long-term research is needed to identify developmental trajectories of psychopathology among different populations after earthquakes.

## Electronic supplementary material

Below is the link to the electronic supplementary material.

Electronic supplementary material 1 (DOCX 36 kb)
