# Supplementary material for: Post-traumatic Stress and Depressive Symptoms Among Adolescents After the 2015 Earthquake in Nepal: A Longitudinal Study
Source: Child Psychiatry Hum Dev. 2021 Feb 19;53(3):430–9. doi: 10.1007/s10578-021-01136-3 (PMC9107405; doi:10.1007/s10578-021-01136-3)
Supplement: Supplementary file 1 — Electronic supplementary material 1 (DOCX 36 kb) [file 10578_2021_1136_MOESM1_ESM.docx]

Supplementary

**Table 1** Demographic and earthquake-related factors of adolescents in PTSS groups

|  | **Resilient**  **n** | **%** | **Recovered**  **n** | **%** | **Delayed**  **n** | **%** | **Chronic**  **n** | **%** | **Total**  **N** | **%** | **P-value ^a^** |
| --- | --- | --- | --- | --- | --- | --- | --- | --- | --- | --- | --- |
| Sex |  |  |  |  |  |  |  |  |  |  | 0.008 |
| Female | 151 | 48.6 | 46 | 61.3 | 25 | 50.0 | 39 | 70.9 | 261 | 53.2 |  |
| Male | 160 | 51.4 | 29 | 38.7 | 25 | 50.0 | 16 | 29.1 | 230 | 46.8 |  |
| Age |  |  |  |  |  |  |  |  |  |  | 0.011 |
| ≤ 15 years | 236 | 75.9 | 54 | 72.9 | 27 | 55.1 | 35 | 63.6 | 352 | 71.9 |  |
| >15 years | 75 | 24.1 | 20 | 27.0 | 22 | 44.9 | 20 | 36.4 | 137 | 28.0 |  |
| Area of location |  |  |  |  |  |  |  |  |  |  | <0.001 |
| Sindhupalchok | 115 | 36.9 | 52 | 69.3 | 36 | 72.0 | 45 | 81.8 | 248 | 50.5 |  |
| Kathmandu | 196 | 63.0 | 23 | 30.7 | 14 | 28.0 | 10 | 18.2 | 243 | 49.5 |  |
| Ethnicity |  |  |  |  |  |  |  |  |  |  | 0.02 |
| Brahmin\Chhetri | 167 | 54.2 | 42 | 56.8 | 20 | 40.0 | 26 | 47.3 | 255 | 52.5 |  |
| Janjati | 133 | 43.2 | 28 | 37.8 | 25 | 50.0 | 22 | 40.0 | 208 | 42.8 |  |
| Dalit | 8 | 2.6 | 4 | 5.4 | 4 | 8.0 | 7 | 12.7 | 23 | 4.7 |  |
| Mother’s education | |  |  |  |  |  |  |  |  |  | 0.872 |
| No education | 76 | 24.5 | 19 | 25.3 | 10 | 20.4 | 17 | 32.1 | 122 | 25.1 |  |
| Secondary | 173 | 55.8 | 39 | 52.0 | 28 | 57.1 | 26 | 49.1 | 266 | 54.6 |  |
| Higher secondary and/or above | 61 | 19.7 | 17 | 22.7 | 11 | 22.5 | 10 | 18.9 | 99 | 20.3 |  |
| Father’s education |  |  |  |  |  |  |  |  |  |  | 0.981 |
| No education | 46 | 14.7 | 9 | 12.0 | 7 | 14.0 | 9 | 16.7 | 71 | 14.5 |  |
| Secondary | 149 | 47.8 | 40 | 53.3 | 24 | 48.0 | 26 | 48.2 | 239 | 48.7 |  |
| Higher secondary and/or above | 117 | 37.5 | 26 | 34.7 | 19 | 38.0 | 19 | 35.2 | 181 | 36.9 |  |
| Pre-earthquake trauma exposure | |  |  |  |  |  |  |  |  |  | <0.001 |
| Yes | 87 | 29.0 | 38 | 52.1 | 29 | 60.4 | 34 | 64.2 | 188 | 39.7 |  |
| No | 213 | 71.0 | 35 | 47.9 | 19 | 39.6 | 19 | 35.9 | 286 | 60.3 |  |
| Trapped/wounded |  |  |  |  |  |  |  |  |  |  | <0.001 |
| Yes | 24 | 7.7 | 14 | 18.7 | 6 | 12.0 | 14 | 25.5 | 58 | 11.8 |  |
| No | 288 | 92.3 | 61 | 81.3 | 44) | 88.0 | 41 | 74.6 | 434 | 88.2 |  |
| House damage |  |  |  |  |  |  |  |  |  |  | 0.031 |
| Not at all | 111 | 35.5 | 16 | 21.3 | 14 | 28.0 | 10 | 18.2 | 151 | 30.6 |  |
| Mild | 93 | 29.7 | 19 | 25.3 | 14 | 28.0 | 17 | 30.2 | 143 | 29.0 |  |
| Moderate (Inhabitable) | 19 | 6.1 | 8 | 10.7 | 5 | 10.0 | 2 | 3.6 | 34 | 6.9 |  |
| Severe (Uninhabitable) | 90 | 28.8 | 32 | 42.7 | 17 | 34.0 | 26 | 47.3 | 165 | 33.5 |  |
| Trauma exposure after earthquake | | |  |  |  |  |  |  |  |  | <0.001 |
| Yes | 74 | 24.5 | 40 | 55.6 | 32 | 68.1 | 38 | 71.7 | 184 | 38.8 |  |
| No | 228 | 75.5 | 32 | 44.4 | 15 | 31.9 | 15 | 28.3 | 290 | 61.2 |  |

Note. PTSS = Posttraumatic Stress Symptoms. ^a^ Pearson Chi-square

Supplementary

**Table 2** Demographic and earthquake-related factors of adolescents in the depressive symptom groups

|  | **Resilient**  **n** | **%** | **Recovered**  **n** | **%** | **Delayed**  **n** | **%** | **Chronic**  **n** | **%** | **Total**  **N** | **%** | **P-value ^a^** |
| --- | --- | --- | --- | --- | --- | --- | --- | --- | --- | --- | --- |
| Sex |  |  |  |  |  |  |  |  |  |  | 0.018 |
| Female | 111 | 49.3 | 50 | 53.8 | 40 | 50.0 | 62 | 68.1 | 263 | 53.8 |  |
| Male | 114 | 50.7 | 43 | 46.2 | 40 | 50.0 | 29 | 31.9 | 226 | 46.2 |  |
| Age |  |  |  |  |  |  |  |  |  |  | 0.001 |
| ≤ 15 years | 173 | 75.9 | 73 | 72.4 | 53 | 67.1 | 51 | 56.1 | 350 | 71.9 |  |
| >15 years | 52 | 23.1 | 19 | 20.7 | 26 | 32.9 | 40 | 43.9 | 137 | 28.1 |  |
| Area of location |  |  |  |  |  |  |  |  |  |  | <0.001 |
| Sindhupalchok | 85 | 37.8 | 47 | 50.5 | 47 | 58.8 | 70 | 76.9 | 249 | 50.9 |  |
| Kathmandu | 140 | 62.2 | 46 | 49.5 | 33 | 41.3 | 21 | 23.1 | 240 | 49.1 |  |
| Ethnicity |  |  |  |  |  |  |  |  |  |  | 0.132 |
| Brahmin\Chhetri | 131 | 58.2 | 43 | 46.2 | 39 | 48.8 | 41 | 45.1 | 254 | 52.4 |  |
| Janjati | 87 | 38.7 | 44 | 47.3 | 36 | 45.0 | 41 | 45.1 | 208 | 42.9 |  |
| Dalit | 6 | 2.7 | 5 | 5.4 | 4 | 5.0 | 8 | 8.8 | 23 | 4.7 |  |
| Mother’s education | |  |  |  |  |  |  |  |  |  | 0.574 |
| No education | 51 | 22.7 | 24 | 25.8 | 20 | 25.0 | 28 | 30.8 | 123 | 25.5 |  |
| Secondary | 128 | 56.9 | 47 | 50.5 | 43 | 53.8 | 40 | 43.9 | 258 | 53.4 |  |
| Higher secondary and/or above | 43 | 19.1 | 22 | 23.7 | 16 | 20.0 | 21 | 23.1 | 102 | 21.1 |  |
| Father’s education |  |  |  |  |  |  |  |  |  |  | 0.664 |
| No education | 30 | 13.3 | 15 | 16.1 | 12 | 15.0 | 15 | 16.5 | 72 | 14.8 |  |
| Secondary | 110 | 48.9 | 49 | 52.7 | 34 | 42.5 | 38 | 41.8 | 231 | 47.4 |  |
| Higher secondary and/or above | 84 | 37.3 | 29 | 31.2 | 34 | 42.5 | 37 | 40.7 | 184 | 37.8 |  |
| Pre-earthquake trauma exposure | |  |  |  |  |  |  |  |  |  | <0.001 |
| Yes | 65 | 28.9 | 31 | 33.3 | 34 | 42.5 | 56 | 61.5 | 186 | 39.4 |  |
| No | 151 | 67.1 | 58 | 62.4 | 45 | 56.3 | 32 | 35.2 | 286 | 60.6 |  |
| Trapped/wounded |  |  |  |  |  |  |  |  |  |  | 0.024 |
| Yes | 20 | 8.9 | 17 | 18.3 | 6 | 7.5 | 16 | 17.6 | 59 | 12.1 |  |
| No | 205 | 91.1 | 76 | 81.7 | 74 | 92.5 | 75 | 82.4 | 430 | 87.9 |  |
| House damage |  |  |  |  |  |  |  |  |  |  | 0.092 |
| Not at all | 81 | 36.0 | 27 | 29.0 | 27 | 33.8 | 16 | 17.0 | 151 | 30.9 |  |
| Mild | 68 | 30.2 | 28 | 30.1 | 21 | 26.3 | 26 | 28.6 | 143 | 29.2 |  |
| Moderate (Inhabitable) | 12 | 5.3 | 6 | 6.5 | 5 | 6.3 | 7 | 7.7 | 30 | 61.4 |  |
| Severe (Uninhabitable) | 64 | 28.4 | 32 | 34.4 | 27 | 33.8 | 42 | 46.2 | 165 | 33.7 |  |
| Trauma exposure after earthquake | | |  |  |  |  |  |  |  |  | <0.001 |
| Yes | 62 | 27.6 | 20 | 21.5 | 44 | 55.0 | 58 | 63.7 | 184 | 38.9 |  |
| No | 157 | 69.8 | 67 | 72.0 | 34 | 42.5 | 30 | 32.9 | 288 | 61.0 |  |

^a^ Pearson Chi-square
